# New South American species of Lamiinae (Coleoptera, Cerambycidae)

**DOI:** 10.3897/zookeys.637.11001

**Published:** 2016-12-02

**Authors:** Maria Helena M. Galileo, Antonio Santos-Silva

**Affiliations:** 1 PPG Biologia Animal, Departamento de Zoologia, Universidade Federal do Rio Grande do Sul, Porto Alegre, RS, Brazil; 2 Museu de Zoologia, Universidade de São Paulo, São Paulo, SP, Brazil; 3 Fellow of the Conselho Nacional de Desenvolvimento Científico e Tecnológico (CNPq)

**Keywords:** Key, Neotropical region, taxonomy

## Abstract

Two new species of cerambycid beetles are described from South America: *Ataxia camiriensis* (Pteropliini), from Bolivia, and *Falsamblesthis uniformis* (Forsteriini), from Peru. The new species are included in previous keys.

## Introduction

Increased Cerambycidae collecting in recent decades, in northwestern South America has led to the discovery of many species new to science. Two of the species are included and described herein.

*Ataxia* Haldeman, 1847 is a relatively large genus of Pteropliini. Currently it includes 41 species, and occurs from the United States of America to southern South America (including Caribbean) (Tavakilian and Chevillotte 2016). Eight species are known from Bolivia (Monné 2016).

*Falsamblesthis* Breuning, 1959 (Forsteriini) includes nine species occurring only in South America (Monné 2016). The new species described here is the first record of the genus for Peru.

## Material and methods

Photographs were taken with a Canon EOS Rebel T3i DSLR camera, Canon MP-E 65mm f/2.8 1–5× macro lens, controlled by Zerene Stacker AutoMontage software. Measurements were taken in ‘‘mm’’ using a micrometer ocular Hensoldt/Wetzlar - Mess 10 in the Leica MZ6 stereomicroscope, also used in the study of the specimen.

The collection acronyms used in this study are as follows:

ACMT
American Coleoptera Museum (James E. Wappes), San Antonio, Texas, USA


FSCA
 Florida State Collection of Arthropods, Gainesville, Florida, USA 


MNKM
 Museo de Historia Natural, Noel Kempff Mercado, Santa Cruz de la Sierra, Bolivia 


SLPC
 Steven W. Lingafelter Private Collection, Hereford, Arizona, USA 


## Results

### Ataxia camiriensis sp. n.

Taxon classificationAnimaliaColeopteraCerambycidae

http://zoobank.org/2E15B030-78CE-45E5-B295-5669AD8E9BC5

Figs 1 , 2 , 3 , 4

#### Diagnosis.

The pronotum without large white pubescence or central band of contrasting pubescence, elytral pubescence not white along suture, and elytral apex widely truncate distinguish this species.

#### Description.

**Female.** Integument dark brown, almost black, except basal half of antennomeres IV–V, basal third of VI, and basal quarter of VII dark reddish brown. Pubescence obscuring nearly all integument.

***Head*.** Frons finely, sparsely punctate; with dense pale golden pubescence interspersed with sparse, long, erect pale yellow and brown setae. Vertex with pale golden pubescence between antennal tubercles and upper eye lobes (this pubescence slightly projected after posterior edge of upper eye lobes), interspersed with long, erect, sparse pale yellow and brown setae; remaining surface with dense greenish-brown pubescence, less dense along coronal suture. Tempora and gena with dense greenish-brown pubescence (more pale yellow depending on angle of light) interspersed with long, erect, sparse pale yellow setae and some thick, brown setae behind lower eye lobe. Submentum with short, decumbent, moderately sparse pale yellow pubescence, denser close to mentum, interspersed with long, erect, sparse pale yellow setae. Labrum with long, decumbent pale yellow and yellowish-brown setae almost obscuring integument, and dense fringe of golden pubescence on distal margin. Distance between upper eye lobes 0.45 times length of scape; distance between lower eye lobes in frontal view 0.75 times length of scape. Antennae 1.55 times elytral length, reaching elytral apex at distal quarter of antennomere IX; scape with narrow apical cicatrix, dorsally and laterally with greenish-brown pubescence interspersed with whitish pubescence, ventrally mostly with whitish pubescence, with long, erect, sparse, thick dark brown setae; pedicel and antennomere III with whitish pubescence except pale yellow pubescence exposing integument on dorsal half (not reaching apex); antennomeres IV–X with whitish pubescence on basal area (covering basal half on IV–V, gradually wider toward X); antennomere XI with whitish pubescence; ventral side of antennomeres with long, erect, white setae on basal half, dark brown on distal half (sparser toward XI); dorsal apex of antennomeres III–X with long, erect dark brown setae (gradually shorter toward X); antennal formula (ratio) based on length of antennomere III: scape = 1.04; pedicel = 0.28; IV = 1.42; V = 1.21; VI = 1.06; VII = 0.95; VIII = 0.85; IX = 0.80; X = 0.74; XI = 0.61.

***Thorax*.** Prothorax 1.3 times wider than long (including lateral tubercles); lateral tubercle conical, with blunt apex, placed at about midlength. Pronotum with 5 gibbosities: one on each side of basal half, subcircular, slightly distinct; one on each side of distal half, subcircular, well-marked; one centrally, elongate, slightly distinct. Pronotal surface coarsely, sparsely punctate; basal half with pale yellow pubescence, with some areas more whitish; distal half with greenish-brown pubescence, more pale yellow on some areas; with long, erect, sparse pale yellow setae and thick dark brown setae on distal half. Sides of prothorax coarsely, sparsely punctate close to pronotum, almost smooth toward ventral side; pubescence as on pronotum. Prosternum with yellowish-white pubescence partially obscuring integument, denser, more pale yellow toward apex of prosternal process. Ventral side of meso- and metathorax with pubescence mostly pale yellow, slightly marmorate with greenish-yellow and yellowish-white pubescence; metasternum with long, erect, sparse pale yellow setae; mesosternal process with tubercle slightly projected. Scutellum with greenish-brown pubescence. *Elytra*. Coarsely, sparsely punctate, more so toward apex; slightly longitudinally sulcate along suture; with low, but distinct carina from apex of basal third to near apex, close to margin of longitudinal sulcus; with two other longitudinal carina, less distinct, between the former and lateral curvature; circum-scutellar region with whitish pubescence; basal 4/5 with greenish-brown pubescence marmorate with pale yellow and yellowish-white pubescence, except lateral area of basal third with pale yellow pubescence (not reaching base) and area on basal declivity and circum-scutellar with white pubescence (more silver on declivity and inconspicuous depending on angle of light); distal fifth with large lateral macula with white pubescence; laterodistal apex with dark brown pubescence; with small, sparse glabrous areas, mainly along suture; with thick, sparse, erect dark brown setae; apex widely truncate. *Legs*. Femora with greenish-brown pubescence, pale yellow ventrally on basal third. Tibiae mostly with greenish-brown pubescence, distinctly golden dorsally on transverse sulcus of mesotibiae and distal area of meso- and metatibiae.

***Abdomen*.** Ventrites with pale yellow pubescence (more green or golden depending on angle of light source), except distal area of ventrite V with dark brown pubescence; sides of ventrite with long, decumbent, sparse pale yellow setae.

***Male*.** It differs from female mainly by the antennae distinctly longer (about 1.8 times longer than elytra), surpassing elytral apex at about midlength of antennomere VIII.

**Figures 1–8.**
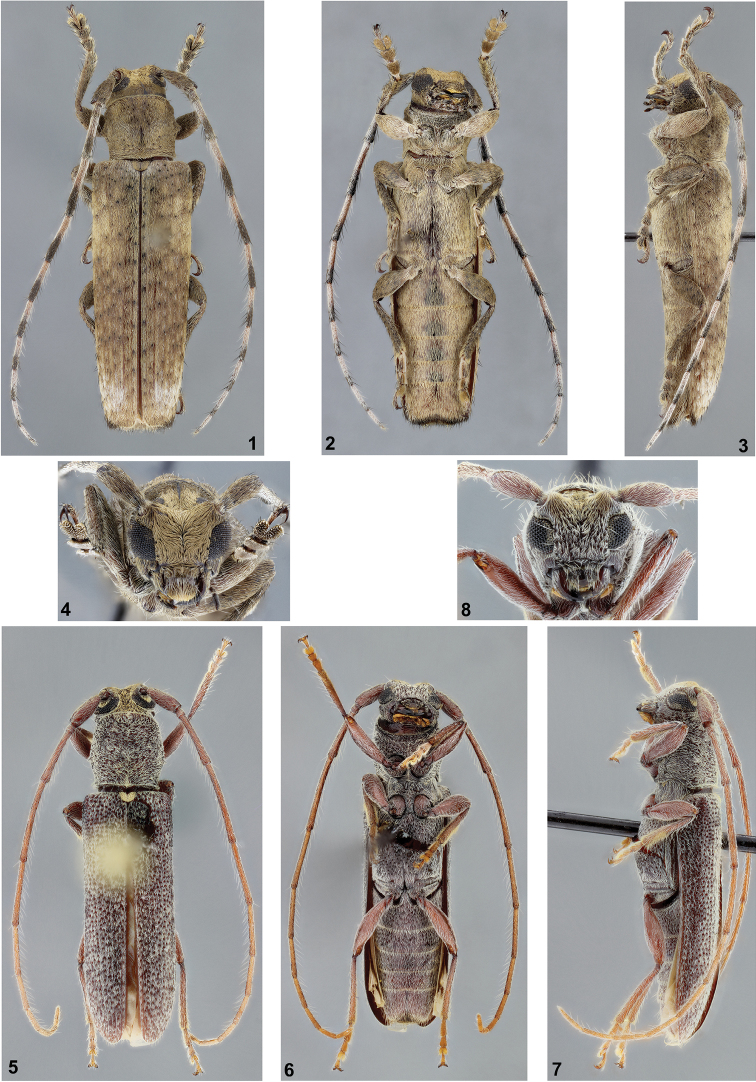
**1–4** *Ataxia camiriensis* sp. n., holotype female: **1** dorsal habitus **2** ventral habitus **3** lateral habitus **4** head, frontal view **5–8** *Falsamblesthis uniformis* sp. n., holotype female: **5** dorsal habitus **6** ventral habitus **7** lateral habitus **8** head, frontal view.

#### Dimensions

**(mm).** Holotype female: Total length, 13.70; prothoracic length, 2.35; basal prothoracic width, 2.55; distal prothoracic width, 2.40; largest prothoracic width (between apices of lateral tubercles), 3.15; humeral width, 3.70; elytral length, 10.10. Paratype male: Total length, 13.00; humeral width, 3.00.

#### Type material.

Holotype female from **BOLIVIA**, Santa Cruz: 20 km N Camiri (road to Eyti, 6–8 km E Hwy 9; 1250 m; 19°5'S / 63°29'W), 26.XI.2013, Wappes & Skillman col. (MNKM). Paratype male from **BOLIVIA**, Santa Cruz: road to Eyti (Cordillera Prov.; 10.5 km NE of Highway 9, 22 km NNE of Camiri; 1140 m; 19°50.56'S / 63°29.05'W), 3–4.XII.2013, Lingafelter col. (SLPC).

#### Etymology.

Named for the city (Camiri) in southern Santa Cruz Department, Bolivia, near where the new species was collected.

#### Remarks.

*Ataxia camiriensis* sp. n. is similar to *Ataxia luteifrons* (Bruch, 1926), but differs as follows: pronotum without large white pubescence areas; elytral pubescence not white along suture; base of elytra without distinct transverse band of white pubescence. In *Ataxia luteifrons* pronotum has a large white pubescent area, elytral pubescence is white along suture, and a distinct transverse band of white pubescence is present along base of elytra.

*Ataxia camiriensis* sp. n. can be included in the alternative of couplet “21” from Breuning (1961):

**Table:** 

21	Elytra coarsely and roughly punctate on basal area. Mexico (Veracruz), Guatemala, Honduras, Nicaragua, Costa Rica, Colombia, Venezuela, French Guiana, Guyana, Peru, Bolivia, Brazil (Amapá, Maranhão)	***Ataxia operaria* (Erichson, 1848)**
–	Elytra very finely punctate on basal area [actually, coarsely, sparsely punctate]	**21**’
21’	Pronotum with large area with white pubescence; base of elytra with transverse band with white pubescence; elytra with white pubescence along suture. Bolivia (Santa Cruz), Paraguay, Argentina (Catamarca, Santiago del Estero, La Rioja, Mendoza, Santa Fé)	***Ataxia luteifrons* (Bruch, 1926)**
–	Pronotum lacking large white pubescence areas; base of elytra without distinct white pubescent transverse band; elytra without white pubescence along suture. Bolivia (Santa Cruz)	***Ataxia camiriensis* sp. n.**

### Falsamblesthis uniformis sp. n.

Taxon classificationAnimaliaColeopteraCerambycidae

http://zoobank.org/4E1D87C5-5C86-49A0-9D1A-96A0E4CBEBBA

Figs 5 , 6 , 7 , 8

#### Diagnosis.

The vertex with yellow pubescence throughout distinguishes this species of the other of the genus.

#### Description.

**Female.** Integument black except mouthparts yellowish-brown, antennae dark reddish-brown, gradually lighter toward distal segments, and legs dark reddish-brown.

***Head*.** Frons moderately coarsely, abundantly punctate; with whitish pubescence partially obscuring integument, more pale yellow toward antennal tubercles; with long, erect, sparse pale yellow setae laterally. Vertex moderately coarsely, abundantly punctate; with yellow pubescence obscuring integument, mainly toward prothorax, interspersed with long, erect, sparse pale yellow setae. Area behind upper eye lobes with pubescence and erect setae as on vertex; area behind lower eye lobes coarsely, sparsely punctate, with yellowish-white pubescence not obscuring integument and long, erect, sparse pale yellow setae close to eye. Area on sides of gulamentum moderately finely rugose-punctate. Gulamentum finely, transversely punctate except smooth elevated anterior area; elevated area with short whitish pubescence, not obscuring integument, interspersed with a few long, erect whitish setae. Postclypeus with yellowish-white pubescence not obscuring integument, centrally interspersed with long, erect, sparse yellowish-white setae. Labrum coplanar with anteclypeus on basal half, inclined on distal half; finely, abundantly punctate laterally, smooth centrally; with decumbent, moderately long yellowish-white setae on punctate area, glabrous centrally; with fringe of yellow setae on distal margin. Distance between upper eye lobes 0.30 times length of scape; distance between lower eye lobes 0.75 times length of scape. Antennae 1.95 times elytral length, reaching elytral apex at apex of antennomere VII; with long, erect, moderately sparse pale yellow seta ventrally (sparser, shorter toward distal segment); antennal formula (ratio) based on length of antennomere III: scape = 0.75; pedicel = 0.15; IV = 1.41; V = 1.12; VI = 0.92; VII = 0.74; VIII = 0.63; IX = 0.60; X = 0.54; XI = 0.54.

***Thorax*.** Prothorax 1.1 times wider than long (including lateral tubercles); lateral tubercle small, conical, with acute apex slightly curved backward, placed before midlength. Pronotum coarsely, densely punctate; with yellowish-white pubescence partially obscuring integument (slightly yellower centrally close to basal and distal margins); with long, erect, sparse pale yellow setae throughout. Sides of prothorax and prosternum with sculpture and pubescence as on pronotum. Mesosternum coarsely, moderately sparsely punctate (punctures slightly smaller than on prosternum); with yellowish-white pubescence not obscuring integument. Mesepisternum, mesepimeron and metepisternum smooth; with yellowish-white pubescence partially obscuring integument. Metasternum coarsely, abundantly punctate laterally, gradually sparser toward center; with yellowish-white pubescence not obscuring integument, sparser toward central region; with short, erect, sparse pale yellow setae throughout. Scutellum with dense yellow pubescence. *Elytra*. Coarsely, abundantly punctate on basal third, slightly finer and sparser toward apex; with yellowish-white pubescence partially obscuring integument; with moderately long, erect, sparse pale yellow setae throughout. *Legs*. Femora with yellowish-white pubescence not obscuring integument. Protibiae with pale yellow pubescence not obscuring integument, except on distal half of ventral side, interspersed with long, erect, sparse pale yellow setae; mesotibiae with pale yellow setae, gradually denser and longer toward apex (notably denser and golden on distal half of dorsal side); metatibiae with pale yellow setae, gradually longer toward apex.

***Abdomen*.** Ventrites finely, moderately sparsely punctate (punctures distinctly finer and sparser from I to V); with yellowish-white pubescence not obscuring integument, interspersed with long, erect, sparse pale yellow setae (more abundant on V); ventrite V with small, longitudinal depression on center of base, with distinct, semicircular depression on distal half (notably deeper centrally close to apex); apex of ventrite V truncate, centrally widely, deeply emarginate.

#### Dimensions

**(mm), holotype female.** Total length, 9.65; prothoracic length, 2.00; basal prothoracic width, 1.75; distal prothoracic width, 1.70; largest prothoracic width (between apices of lateral tubercles), 2.15; humeral width, 2.40; elytral length, 6.70.

#### Type material.

Holotype female from **PERU**, Amazonas: 12 km W Bagua Grande (-5.7257 / -78.5365; 540 m), 14–17.XI.2007, M. E. Irwin & P. D. Parker col. (FSCA).

#### Etymology.

Named for its uniform pubescent appearance.

#### Remarks.

*Falsamblesthis uniformis* sp. n. is similar to *Falsamblesthis ibiyara* Marinoni, 1978, but differs as follows: pronotum convex; distance between upper eye lobes about 1.5 times width of one lobe; pubescence on vertex yellow throughout. In *Falsamblesthis ibiyara* the pronotum is flat, distance between upper eye lobes is wider than twice the width of one lobe, and the pubescence between antennal tubercles and remaining surface of vertex has different color.

*Falsamblesthis uniformis* sp. n. can be included in the alternative of couplet 5 from Galileo and Martins (1987):

**Table:** 

5	Sides of metasternum coarsely and abundantly punctate	**5**’
–	Sides of metasternum smooth. Ecuador	***Falsamblesthis macilenta* (Gounelle, 1910)**
5’	Distance between upper eye lobes about 1.5 times width of one lobe; vertex with yellow pubescence throughout. Peru	***Falsamblesthis uniformis* sp. n.**
–	Distance between upper eye lobes wider than twice the width of one lobe; pubescence between antennal tubercles and remaining surface of vertex with different color. Brazil (Bahia, Minas Gerais, Espírito Santo, Rio de Janeiro, São Paulo, Paraná, Santa Catarina, Rio Grande do Sul)	***Falsamblesthis ibiyara* Marinoni, 1978**

## Supplementary Material

XML Treatment for Ataxia camiriensis

XML Treatment for Falsamblesthis uniformis
